# 

**Published:** 1910-12-24

**Authors:** 


					Appointments and Resignations.
Mr. W. R. Douglas, F.R.C.S., has been elected
honorary surgeon to the Ancoats Hospital, Manchester.
Mr. Garnett Wright, F.R.C.S., has resigned his post
as honorary surgeon to the Ancoats Hospital.
Dr. Buchanan Ross, of Cawood, near Selby, has been
appointed medical officer for the Cawood and Riccalf
district by the Selby Board of Guardians.
Mr. E. W. Adams, M.D. (London and Sheffield), has
been appointed to the post of Lecturer in Materia Medica
and assistant to the Professor of Materia Medica, Pharma-
cology, and Therapeutics, Sheffield University.
Obituary.
Dr. James Edward Pollock, Physician Extraordinary
to Queen Victoria, died last week in London in his ninety-
third year. He took his M.R.C.P. in 1850, and became a
Fellow of the College in 1864. He had studied at the
University of Aberdeen, where he graduated M.D. in
1850. Among other appointments he held that of consult-
ing physician to the Brompton Hospital for Consumption.
Dr. Pollock was a contributor to the medical journals on
consumption.
The death is announced at Bournemouth of Surgeon-
General John Meane, aged eighty-two. He took his
M.R.C.S. in 1850, and joined the Army Medical Depart-
ment in 1852. He went through the Crimean war, the
Afghan war of 1878 to 1880, and retired in 1881 with
the honorary rank of deputy surgeon-general. He re-
ceived the Turkish and Afghan medals.
Gifts and Bequests.
Mr. Edward Pritchard Martin, of The Hill, Aber-
gavenny, a past-president of the Iron and Steel Institute,
who left estate of the gross value of ?236,249 Os. 3d., has
bequeathed ?500 to the Merthyr and Dowlais Hospital.
Lady Juliet Duff has received the following further con-
tributions towards the fund for freeing Charing Cross*
Hospital from its mortgage debt : Lord Iveagh, ?100;
Messrs. Cox and Co., ?52 10s.; Messrs. 'Hoare, ?50;
Messrs. Jaeger, Ltd., ?21; Mr. W. S. M. Burns, ?10 10s. j
Mr. Meyer Sassoon, ?10; Sir Francis Montefiore, Bt., ?10;
Colonel Baldock, C.B., ?10; Miss Lomax, ?10: Gertrude
Lady Penrhyn, ?5 5s.; Messrs. Woodhead and Co., ?5 5s,
The Hon. Treasurer of Ancoats Hospital, Manchester,
has received.?50 from Mr. J. N. Field, Bowden. Cheshire,
also ?25 from Mrs. Field.
Jottings.
The annual Christmas entertainment at the German
Hospital, Dalston, will take place to-day (Saturday) at
3.30 p.m. As usual, the wards will be decorated and lit
up; there will be carol singing by the Sisters and others,
and a distribution of presents to the older patients and of
toys to the little ones. For the enjoyment of these lattei-
a special electrically lighted Christmas tree has been pro-
mised by the Hackney Electricity Works. About 14a
patients will participate in the festivities.
For some time past the accommodation at the Anti-
Vivisection Hospital, Battersea has been stretched to its-
utmost limits, and great difficulty has been experienced
with respect to the in-patients. Through the generosity of
a lady supporter of the hospital, Miss Tracey, an adjoin-
ing house has been acquired which will enable the hospital
to have more accommodation for children and others.
The Queen has sent a special donation to the Royal
Hospital for Incurables, Putney Heath, with a command
that the sum shall be devoted " to any special celebration of
Christmastide that may be contemplated at the Royal Hos-
pital," and a further wish to the effect that Her Majesty
will be " glad to know that hor gift has helped to make-
Christmas pass more pleasantly among the inmates of the
Hospital." The Queen, who is patron of this National
Charity, has also sent a gift of pheasants for the in-
patients.
Princess Christian attended a viotmcc perfornianco
by the Windsor Strollers of "Under the Red Robe," in
the New Theatre at Windsor, last week. The Strollers
gave performances of " The Marriage of Kitty " and a one-
act play, "The Sentence," by Mr. Harold Whitaker.
Among those taking part in these performances were Lady
Alington, Mrs. Charles Crutchley, the Hon. Stephen Powys,
Sir Spencer Ponsonby-Fane, Colonel Chater, and Lieut.-
Colonel Newnham-Davis. The profits will be given to the
King Edward VII. Hospital at Windsor.
Princess Alexis Dolgorouki lent her house, in Upper
Grosvenor Street, recently for an exhibition of amateur-
art (portraits by Miss Maud Worsfold) in aid of the-
Children's Sanatorium at Holt, Norfolk.
The fund for paying off the debt of Cardiff Infirmary
has reached over ?7,000 since the appeal was issued a
fortnight ago.
The Christmas entertainment for the patients of the
Belgrave Hospital for Children, Clapham Road, S.W., will
be held at the hospital (3.30 to 6), on Wednesdav. January 4,
1911.
THE IDEAL WORK OF REFERENCE.
BURDETT'S HOSPITALS & CHARITIES.
1910 EDITION.
The Year-Book of Philanthropy and Hospital Annual,
Edited by SIR HENRY BURDETT, K.C.B., K C.V.O.
A Complete Guide and Directory of the Hospitals, Asylums, and Charitable Institutions throughout the English-speaking World.
Contains Special New Chapters on:?Voluntary Hospitals in 1909.?Soundness of the Voluntary System.?The need of
greater zeal on the part of those who accept office.?State-aided Hospitals in the U.S.A. and Canada.?The Appropriation of
Public Funds for the partial Support of Voluntary Hospitals in U.S.A. and Canada.?The Need of an Apostle and of greater
interest on the part of the Clergy, &c., &c.
TlFEXTT-FlIiST YEAR OF PUBLICATIOX.
Over 1,000 pages. Price 10/S net; Post Free, 10/11.
ORDER your copy of the Medical Whitaker NOW.
Publishers : THE SCIENTIFIC PRESS, Ltd, 28 k 29 Southampton Streat, Strand, London, W.C.
GENERAL PRACTITIONERS'
CONTRIBUTIONS.
The Hospital devotes a special page to General
Practitioners' contributions. We therefore invite
from practitioners contributions based upon their
.experience in the management of cases, and in the
treatment and diagnosis of disease; especially shall
?we be prepared to welcome articles dealing, practi-
cally, with treatment, and with the use and value
?of new remedies and methods.
No article should exceed 1,100 words, and, if
accepted, one guinea for a contribution of this
length will be paid to the writer after publication.
Each communication should be accompanied by a
-stamped directed envelope for the return of the MS.
if found unsuitable.
Contributions should be written, or preferably
typed, on one side of the paper only, and all articles
sent in are accepted upon the distinct understanding
that they are forwarded to The Hospital only.
Books for Review.
Publishers are particularly requested to send
advance proofs of any new books of importance,
whenever possible, as the Editor has made arrange-
ments to publish immediate reviews, and on a new
plan
The Relaxations of Medical Men.
We shall also be glad to pay for accepted contri-
butions, from any member of the profession, on the
subject of the relaxations of practitioners. This
opens up a wide field, as it includes natural history,
photography, sport, indoor recreations, and motor-
ing. Whenever possible, original illustrations and
photographs should be sent with the MS.
BUSINESS NOTICES.
Annual Subscriptions.
The rate of annual subscriptions to The
Hospital, either direct from the Office or through
agents, is:-^-
For the United Kingdom  15s. a year.
For the Colonies and Abroad ... 17s. ,,
For the United Kingdom (with
Insurance Policy)  ?1 ,,
inclusive of postage in each case.
Letters relating to the Publishing, Sale and
Advertisement Departments must be addressed to
the Manager (not to the Editor): ?
The Manager,
The Hospital Building,
28 and 29 Southampton Street,
Strand, London, W.C.
Just Issued. Ninth Edition, Rewritten and Enlarged, 9s. net.
PHARMACY, MATERIA MEDICA & THERAPEUTICS.
Being a Commentary on the B.P. and its Drugs, with their Preparations,
and on all the new Non-official Remedies in use, with Chapters on the
different processes used in Dispensing, Prescription-writing, Latin
Glossary, &c. By Sip W. WHITLA, M.D., LL.D., Professor, Materia
Medica, Queen's University, Belfast, and Senior Physician, Royal Victoria
Hospital.
By the same Author. New Work. 1,900 pages. Orown8vo.25s.net.
PRACTICE OF MEDICINE.
Containing in two Handy Volumes a complete and independent Article on
the Etiology, Symptoms, and Diagnosis of every Medical Disease; arranged
in Dictionary Form for Practitioners and Students.
London: Bailli&re, Tindall & Cox, 8 Henrietta Street, W.O.

				

